# Complete Genome of Isoprene Degrading *Nocardioides* sp. WS12

**DOI:** 10.3390/microorganisms8060889

**Published:** 2020-06-12

**Authors:** Lisa Gibson, Nasmille L. Larke-Mejía, J. Colin Murrell

**Affiliations:** School of Environmental Sciences, University of East Anglia, Norwich NR4 7TJ, UK; lisa.gibson@uea.ac.uk (L.G.); N.Mejia@uea.ac.uk (N.L.L.-M.)

**Keywords:** isoprene, isolate, genome, degradation, *Nocardioides*, rubber

## Abstract

Isoprene is a climate-active gas whose wide-spread global production stems mostly from terrestrial plant emissions. The biodegradation of isoprene is carried out by a number of different bacteria from a wide range of environments. This study investigates the genome of a novel isoprene degrading bacterium *Nocardioides* sp. WS12, isolated from soil associated with *Salix alba* (Willow), a tree known to produce high amounts of isoprene. The *Nocardioides* sp. WS12 genome was fully sequenced, revealing the presence of a complete isoprene monooxygenase gene cluster, along with associated isoprene degradation pathway genes. Genes associated with rubber degradation were also present, suggesting that *Nocardioides* sp. WS12 may also have the capacity to degrade poly-cis-1,4-isoprene.

## 1. Introduction

Isoprene (2-methyl-1,3-butadiene) is a major biogenic volatile compound (BVOC) with atmospheric emissions of 400–600 Tg y^−1^, making it similar in scale to methane [1,2]. As a climate-active gas, it plays a wide and varied role in the Earth’s atmospheric chemistry. With an atmospheric lifetime ranging from 0.8 h to 1.3 days, it is highly reactive and susceptible to attack by hydroxyl, nitrate and ozone radicals [3]. When encountering high concentrations of NOx (such as in polluted urban environments) the products of isoprene oxidation by hydroxyl (OH) radicals contribute to an increase of ozone levels in the troposphere, which in turn has a deleterious effect on air quality. In less polluted environments that have lower levels of NOx, isoprene can interact with ozone directly, and thus lower atmospheric ozone concentrations whilst recycling OH [1,4]. Isoprene can also lead to an increase in the lifetime of methane in the atmosphere, contributing to global warming [4]. The oxidation products of isoprene can lead to the production of secondary organic aerosols (SOCs) [5,6]. This is another important factor when considering isoprene’s role in atmospheric chemistry, as the scale of isoprene emissions globally results in a significant contribution to total SOC production, a process that acts as a source of cloud condensation nuclei and thus contributes to global cooling [5,6]. As such, the nuances of the net effect of isoprene on global temperature shifts are particularly susceptible to local atmospheric conditions and the effects of isoprene on the Earth’s climate are also predicted to change significantly in the future as a result of changing land use and climate change [7].

As a climate-active gas, isoprene is unusual in that the vast majority, approximately 90%, of its production is by terrestrial plants [2]. Terrestrial emissions of isoprene show a high temporal and geographic variation that is dependent on climate, temperature fluxes and local vegetation types [8]. Specific plant species act as another variable in isoprene production, with emissions fluctuating significantly between even closely related species, for example, while American oaks are considered high emitters, there are many European oaks that do not synthesize isoprene at all [9,10,11]. Willow trees (*Salix alba*) such as the one that harboured *Nocardioides* sp. WS12, the focus of this study, are considered high emitters. For example, isoprene emissions of 64.6 nmol m^−2^ s^−1^ in the canopy of willow forests have been observed [12,13].

While the production and atmospheric fate of isoprene has been well studied, biological consumption in the isoprene biogeochemical cycle remains relatively unexplored. Field chamber and continuous-flow studies have shown that soils are a biological sink for isoprene at environmentally relevant concentrations [14,15,16]. Several bacterial strains capable of growing on isoprene as a sole carbon and energy source have been isolated from soil, phyllosphere and aquatic environments (reviewed in [17]). All characterised isoprene-utilising microorganisms contain six genes (*isoABCDEF*) that encode the isoprene monooxygenase (IsoMO) enzyme, which catalyses the first step in the isoprene degradation pathway. Adjacent genes *isoGHIJ* encode enzymes involved in the subsequent steps of isoprene metabolism [18,19,20]. The IsoMO belongs to the soluble diiron monooxygenase (SDIMO) family [21] and the α-subunit contains the diiron centre at the putative active site.

The genus *Nocardioides* belongs to the family *Nocardioidaceae* within the suborder *Propionibacterineae*. Members of the genus *Nocardioides* are Gram-positive and non-acid-fast, catalase-positive, aerobic and mesophilic nocardioform actinomycetes [22]. *Nocardioides* spp. have been found in the phyllosphere environment of a number of different plant species [13,23,24,25], although, until recently, none that have the ability to degrade isoprene have been isolated. However, Actinobacteria such as *Nocardioides* are one of the most diverse, well characterized and metabolically versatile groups of microorganisms. Actinobacteria are well represented amongst previously isolated isoprene-degrading bacteria, such as the most extensively characterised isoprene degrader, *Rhodococcus* AD45 [18,19,20], along with others, such as *Gordonia* and *Mycobacterium* [26,27].

Previously [13], a novel isoprene-degrading bacterium, *Nocardioides* sp.WS12, was isolated from the soil associated with a willow tree. In this study, its genome was sequenced, revealing a full isoprene metabolic gene cluster and the potential for rubber degradation.

## 2. Materials and Methods

### 2.1. Isolation and Growth

The isolation of *Nocardioides* sp. WS12 from soil samples collected 10–20 cm from the trunk of a willow tree (*Salix alba*) and 5–10 cm below the soil surface was carried out as previously described [13]. Subsequent cultures were maintained in Ammonia Nitrate Mineral Salts (ANMS) media (adapted from Brenner et al. [28] with the addition of 5 gL^−1^ ammonium and nitrate), supplemented with 125 ppmv of isoprene and incubated at 25 °C with shaking. Under these conditions, *Nocardioides* sp. WS12 displayed a specific growth rate of 0.033 h^−1^ with a generation time of 21 h, reaching a max OD_540_ of 1.0. *Nocardioides* sp. WS12 could grow under isoprene concentrations of up to 250 ppmv without any significant impact on growth.

### 2.2. Genome Sequencing

After growth on isoprene and confirmation of purity through plating cultures to complex media and visual microscopic analysis, *Nocardioides* sp. WS12 cells were grown at 25 °C using 10 mM glucose and plated to R2A (Oxoid) agar plates. After 3 days of growth, biomass was collected from plates and deposited into barcoded bead tubes supplied by MicrobesNG (University of Birmingham, Birmingham, UK). Combined long-read and short-read genome sequencing was conducted by MicrobesNG as follows: For Illumina sequencing, beads were washed with extraction buffer containing lysozyme and RNase A and incubated for 25 min at 37 °C. Proteinase K and RNaseA were added and incubated for 5 min at 65 °C. Genomic DNA was purified using an equal volume of Solid Phase Reversible Immobilisation (SPRI) beads and resuspended in EB buffer. DNA was quantified in triplicates with the Quant-It dsDNA High Sensitivity assay in an Eppendorf AF2200 plate reader. Genomic DNA libraries were prepared using a Nextera XT Library Prep Kit (Illumina, San Diego, CA, USA) following the manufacturer’s protocol with the following modifications: two nanograms of DNA instead of one were used as input, and the PCR elongation time was increased to 1 min from 30 s. DNA quantification and library preparation were carried out on a Hamilton Microlab STAR automated liquid handling system. Pooled libraries were quantified using the Kapa Biosystems Library Quantification Kit for Illumina on a Roche light cycler 96 qPCR machine. Libraries were sequenced on the Illumina HiSeq using a 250bp paired end protocol.

Long-read sequencing was carried out as follows. Broth cultures were pelleted out and the pellet was resuspended in the cryoperservative of a Microbank™ (Pro-Lab Diagnostics UK, Wirral, UK) tube and stored in the tube. Approximately 2 × 10^9^ cells were used for high molecular weight DNA extraction using a Nanobind CCB Big DNA Kit (Circulomics, Baltimore, MD, USA). DNA was quantified with the Qubit dsDNA High Sensitivity assay in a Qubit 3.0 (Invitrogen) Eppendorf UK Ltd., Loughborough, UK). Long-read genomic DNA libraries were prepared with Oxford Nanopore SQK-RBK004 Kit with Native Barcoding EXP-NBD104/114 (ONT, Oxford, UK) using 400–500 ng of high molecular weight DNA. Twelve barcoded samples were pooled together into a single sequencing library and loaded in a FLO-MIN106 (R.9.4 or R.9.4.1) flow cell in a GridION (ONT, Oxford, UK). Reads were adapter trimmed using Trimmomatic 0.30 [29] with a sliding window quality cutoff of Q15. Combined genome assembly was performed with Unicycler v0.4.0 [30].

### 2.3. Genome Analysis and Comparison

Genome quality was assessed with The MicroScope Microbial Genome Annotaion and Analysis Platform version 3.13.5 (https://mage.genoscope.cns.fr/microscope) [31]. This platform was used to determine the general characteristics of the Nocardioides sp. WS12 genome and to query the presence of genes of interest. Amino acid sequences that were likely candidates for enzymes involved in the isoprene degradation pathway were compared to a personally curated database of such proteins with the use of tBLASTn (https://blast.ncbi.nlm.nih.gov/Blast.cgi) [32]. The Microbial Genome Atlas (MiGA) (http://microbial-genomes.org) [33] was used to determine taxonomic affiliation, novelty and gene diversity with the use of the National Centre for Biotechnology Information (NCBI) prokaryotic genome database. Genomic data generated in this study were deposited to the National Centre for Biotechnology Information (NCBI) Prokaryotic Genome Database under Bioproject PRJNA272922 (Biosample SAMN15030414).

## 3. Results and Discussion

### 3.1. Genome Sequencing and Identification

A complete, closed genome was retrieved for *Nocardioides* sp. WS12, consisting of a single contig of 5.2 Mb. This places *Nocardioides* sp. WS12 at the larger range of genome sizes for its genus, with the average at about 4Mbp and some, such as *Nocardioides nitrophenolicus*, isolated from industrial wastewater, being significantly smaller at under 2Mbp [34]. GC-content was average for the genus at 69% (Table 1). No plasmids were detected in *Nocardioides* sp. WS12.

*Nocardioides* sp. WS12 contains two 16S rRNA genes that were both identified as *Nocardioides* at an identity of 100%, though at the species level they diverged, with the first sharing 97.58% nucleic acid identity with *Nocardioides* sp. strain DK7869 (unpublished) and the second sharing 96.96% nucleic acid identity with the *Nocardioides aromaticivorans* strain H-1 [35]. Taxonomic analysis of the entire genome of *Nocardioides* sp. WS12 revealed that it had an Average Amino Identity (AAI) of 83.34% with *Pimelobacter simplex* [36] and 82.26% with *Nocardioides humi* [37]. This is far below the 95% threshold for shared species identity, and, as such, it is likely *Nocardioides* sp. WS12 belongs to a species not currently represented by the 115 currently available *Nocardioides* genomes in the NCBI prokaryotic database.

### 3.2. Isoprene Degradation Gene Cluster

**Figure 1:**
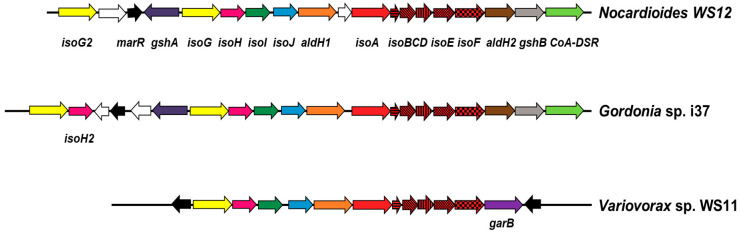
Isoprene degradation gene cluster of *Nocardioides* sp. WS12 compared to the corresponding gene clusters of another Actinobacterial isoprene degrader, *Gordonia* sp. i37 and the Gram-negative isoprene degrader *Variovorax* sp. WS11.

The presence and arrangement of genes associated with isoprene degradation in *Nocardioides* sp. WS12 follow a very similar structural organisation to that of the previously isolated Actinobacterium, *Gordonia* sp. i37 [27]. That is, a complete isoprene monooxygenase gene cluster (*isoABCDEF*) is adjacent to an aldehyde dehydrogenase gene (*aldH2*), a glutathione synthase gene (*gshB*), and a CoA-disulfide reductase gene (*CoA-DSR*). Upstream of the isoprene monooxygenase, *Nocardioides* sp. WS12 contains *isoGHIJ*, which encode a putative coenzyme A transferase, a dehydrogenase and two glutathione transferases involved in the downstream steps of the isoprene degradation pathway. The genome of *Nocardioides* sp. WS12, like that of *Gordonia* sp. i37, contains a second copy of a glutathione synthase gene (*gshA*) and the putative transcriptional regulator *marR*, along with a duplicate copy of *isoG* (*isoG2*). However, the *Nocardioides* sp. WS12 genome differs in that it does not contain a duplicate of *isoH* that is found in *Gordonia* sp. i37 (Figure 1).

As with other Gram-positive Actinobacteria previously shown to degrade isoprene, the isoprene degradation gene cluster of *Nocardioides* sp. WS12 shows some distinct differences to those of Gram-negative isoprene degraders, such as *Variovorax* sp. WS11 (Figure 1), which does not contain duplicates of any of the downstream isoprene degradation genes and contains the gene *garB* encoding a glutathione reductase, which is not present in *Nocardioides* sp. WS12. When polypeptides encoding for *iso* genes were compared to those of other bona-fide isoprene degraders, each showed >50% amino acid identity with isoprene metabolism enzymes from other Actinobacteria, such as *Gordonia, Rhodococcus* and *Mycobacterium* (Table 2).

### 3.3. Rubber Degradation

Actinobacteria are well represented amongst those bacteria known to degrade rubber (poly-cis-1,4-isoprene). Examples include *Streptomyces, Actinoplanes, Gordonia, Mycobacterium* and *Micromonospora* [39]. There are three main types of rubber oxygenases. The first is RoxA, which has mainly been found in Gram-negative rubber-degrading bacteria and was first identified in *Xanthomonas* sp. 35Y [40]. The second, Lcp, is a latex-clearing protein, so called for the ability of some bacteria expressing the protein to form clearing zones when plated onto solid media containing natural latex. The third rubber oxygenase type, RoxB, is also found in Gram-negative bacteria, such as *Xanthamonoas* sp. strain 35Y, *Haliangium ochraceum, Myxococcus fulvus*, and *Corallococcus coralloides*, and is loosely related to RoxA [41].

A gene coding a protein from the second group of rubber oxygenases, *lcp*, was found in *Nocardioides* sp. WS12. Adjacent to this *lcp* gene was a gene encoding a putative transcriptional regulator of the Tet-R family, which is also frequently found to be associated with *lcp* in rubber-degrading bacteria [42]. The Lcp of *Nocardioides* sp.WS12 shared a 55% amino acid identity with the Lcp from both *Streptomyces* K30 and *Gordonia polyisoprenivorans*, both of which have been shown to degrade rubber, though via two different mechanisms [43,44]. The main industrial source of isoprene in the environment is due to the production of synthetic rubber [45], and previous studies have identified the presence of *isoA*-containing bacteria in soil from rubber tyre dump environments [46]. It is perhaps unsurprising that a bacterium such as *Nocardioides* sp. WS12 that is adapted to degrade the monomer of rubber, isoprene, would also be adapted to the degradation of rubber itself.

## 4. Conclusions

The complete genome sequence of *Nocardioides* sp. WS12 provides valuable insights into the metabolic capabilities of a novel isoprene degrading Actinobacterium. Details of the isoprene degradation gene cluster further contribute to our understanding of the differences between Gram-positive and Gram-negative isoprene-degrading bacteria and provide new isoprene degradation gene sequence data to inform robust searches for isoprene degraders in the environment in the future. The presence of genes associated with rubber degradation in *Nocardioides* sp. WS12 suggests a potentially interesting link between isoprene and polyisoprene biodegradation, with scope for future investigation and possible biotechnological applications.

## Figures and Tables

**Table 1:** Characteristics of the genome of *Nocardioides* sp. WS12.

Length (bp)	5,171,066
Undetermined bases	0
GC (%)	68.66
Contigs	1
N50	5,171,066
Predicted Proteins	4975
Ave. Length (aa)	323
Coding Density (%)	93.3
Completeness	99.23%
Contamination	0.52%
Pseudogenes	2
tRNA types	21
Total tRNAs	52

**Table 2:** Blastp comparison of polypeptides encoded by isoprene degradation genes in *Nocardioides* sp. WS12 to those from isoprene degrading Actinobacteria.

Polypeptide	Closest *bona-fide* Isoprene Degrader	References	Coverage %	(aa) ID%
IsoA	*Gordonia polyisoprenivorans* strain i37 IsoA	[26,27]	99	85.19
IsoB	*Rhodococcus opacus* strain PD630 IsoB	[18,38]	100	57.95
IsoC	*Mycobacterium* sp. strain AT1 IsoC	[27]	100	65.77
IsoD	*Rhodococcus* sp. AD45 IsoD	[19]	100	67.3
IsoE	*Rhodococcus opacus* strain PD630 IsoE	[18,38]	100	62.96
IsoF	*Gordonia polyisoprenivorans* strain i37 IsoF	[26,27]	99	52.52
IsoG	*Rhodococcus opacus* strain PD630 IsoG	[18,38]	100	76.56
IsoH	*Rhodococcus* sp. AD45 IsoH	[19]	100	73.45
IsoI	*Rhodococcus* sp. strain WS4 IsoI	[13]	100	67.23
IsoJ	*Rhodococcus* sp. strain WS4 IsoJ	[13]	100	69
AldH1	*Gordonia* sp. strain OPL2 AldH1	(in prep)	98	65.3
IsoG2	*Rhodococcus* sp. strain WS4 IsoG	[13]	96	59.64
